# Spin transport properties in a topological insulator sandwiched between two-dimensional magnetic layers

**DOI:** 10.1038/s41598-024-80694-7

**Published:** 2025-01-17

**Authors:** Nezhat Pournaghavi, Banasree Sadhukhan, Anna Delin

**Affiliations:** 1 Department of Applied Physics, School of Engineering Sciences, KTH Royal Institute of Technology, AlbaNova University Center, SE-10691 Stockholm, Sweden; 2 Department of Physics and Astronomy, Uppsala University, 516, SE-75120 Uppsala, Sweden; 3 Department of Physics, University of Texas at Austin, Austin, TX 78712 USA; 4 Department of Physics and Nanotechnology, SRM Institute of Science and Technology, Kattankulathur, Chennai, Tamil Nadu 603203 India; 5 Tata Institute of Fundamental Research, Hyderabad, Telangana 500046 India; 6 Swedish e-Science Research Center (SeRC), KTH Royal Institute of Technology, SE-10044 Stockholm, Sweden; 7 Wallenberg Initiative Materials Science for Sustainability (WISE), KTH Royal Institute of Technology, SE-10044 Stockholm, Sweden

**Keywords:** Electronic properties and materials, Magnetic properties and materials, Spintronics, Surfaces, interfaces and thin films, Topological matter

## Abstract

Non-trivial band topology along with magnetism leads to different novel quantum phases. When time-reversal symmetry is broken in three-dimensional topological insulators (TIs) through, e.g., the proximity effect, different phases such as the quantum Hall phase or the quantum anomalous Hall(QAH) phase emerge, displaying interesting transport properties for spintronic applications. The QAH phase displays sidewall chiral edge states, which leads to the QAH effect. We have considered a heterostructure consisting of a TI, namely Bi$_2$Se$_3$, sandwiched between two two-dimensional ferromagnetic monolayers of CrI$_3$, to study how its topological and transport properties change due to the proximity effect. Combining DFT and tight-binding calculations, along with non-equilibrium Green’s function formalism, we show that a well-defined exchange gap appears in the band structure in which spin-polarised edge states flow. In a finite slab, the nature of the surface states depends on both the cross-section and thickness of the system. Therefore, we also study the width and finite-size effects on the transmission and topological properties of this magnetised TI nanoribbon.

## Introduction

Three dimensional topological insulators (3D TIs) are characterized by an insulating gap in the bulk and gapless 2D surface states at the termination surfaces, which are energetically positioned inside the bulk gap and topologically protected by time-reversal symmetry (TRS)^1–4^. In the presence of TRS, the topological surface states consist of spin-momentum locked states forming a single massless Dirac cone, whose existence is guaranteed by the bulk $\mathcal{Z}_2$ topological invariant^5^. In this system, at each edge, helical states with opposite spin propagate in opposite directions. Introducing magnetic order in TIs breaks TRS and results in different topological phases. TRS can be broken for instance through an external magnetic field – leading to the quantum Hall effect (QHE) – or by introducing spontaneous magnetization in the system, which leads to the quantum anomalous Hall effect (QAHE)^6^.

When TRS is broken at the surface of a TI, a half-integer quantum anomalous Hall conductivity $\pm e^2/2h$ (*h* is the Planck constant and *e* is the electron charge) arises at that surface^7–10^. In a TI thin film in a Hall bar geometry, the resulting topological state depends on how TRS is broken at the two surfaces. When the magnetization at the top and the bottom surfaces point in the same direction and the chemical potential is inside the exchange gap, the system is in the Chern insulator (CI) state. This phase is characterized by a non-zero integer Chern number $\mathcal C$ and displays the QAHE. Due to the emergence of chiral edge states on the film side walls, the Hall conductance is quantized $\sigma _\textrm{H} = \mathcal{C} e^2/h$^11–14^.

One approach to generate magnetism at the surfaces of a TI thin film is to exploit the interfacial proximity with an adjacent film of a magnetic insulator or semiconductor^15–17^. This method is favorable over doping the system with magnetic impurities, since the properties of the topological system will be less modified. Some crucial issues here are the selection of a proper magnetic material, and the nature of their coupling with the TI film. The magnetic layer should be able to generate a sizable exchange gap; at the same time, the interface hybridization should not be too strong, since we want to to avoid damaging the Dirac surface states or shifting them away from the Fermi level. For this purpose, over the past few years, several magnetic TI heterostructures have been proposed theoretically and realized experimentally^18–24^. The majority of these consist of ferromagnetic (FM) insulators, but a few examples with antiferromagnetic (AFM) materials have been considered^15,16,25–28^. Despite all this effort, realization of the CI in these heterostructures is still quite challenging. There are several reasons for this issue, however the finite-size effect is one of the main factors that should be taken into account when studying this phase.

In this work, we employ density functional theory (DFT) along with a tight-binding model and a ${\varvec{k}}\cdot {\varvec{p}}$ model to thoroughly study the electronic and topological properties of an FM/TI/FM trilayer heterostructure, where the 2D ferromagnetic material CrI$_3$ is used to magnetize the Dirac surface states of Bi$_2$Se$_3$^29^. CrI$_3$ is a 2D van der Waals ferromagnetic monolayer with high Curie temperature (45 K)^29,30^, thus, it is a very promising candidate for magnetizing the surface states of Bi$_2$Se$_3$ without destroying their topological character. The goal of this work is to investigate the physical conditions under which the emergence of edge states in the Chern insulator phase can be detected. Since the size of the studied system is very large, a DFT-based investigation such as the present one is computationally very demanding. We therefore also propose and investigate substitution methods that will be useful also generally for other large systems.

## Method

### System geometry

**Fig. 1:**
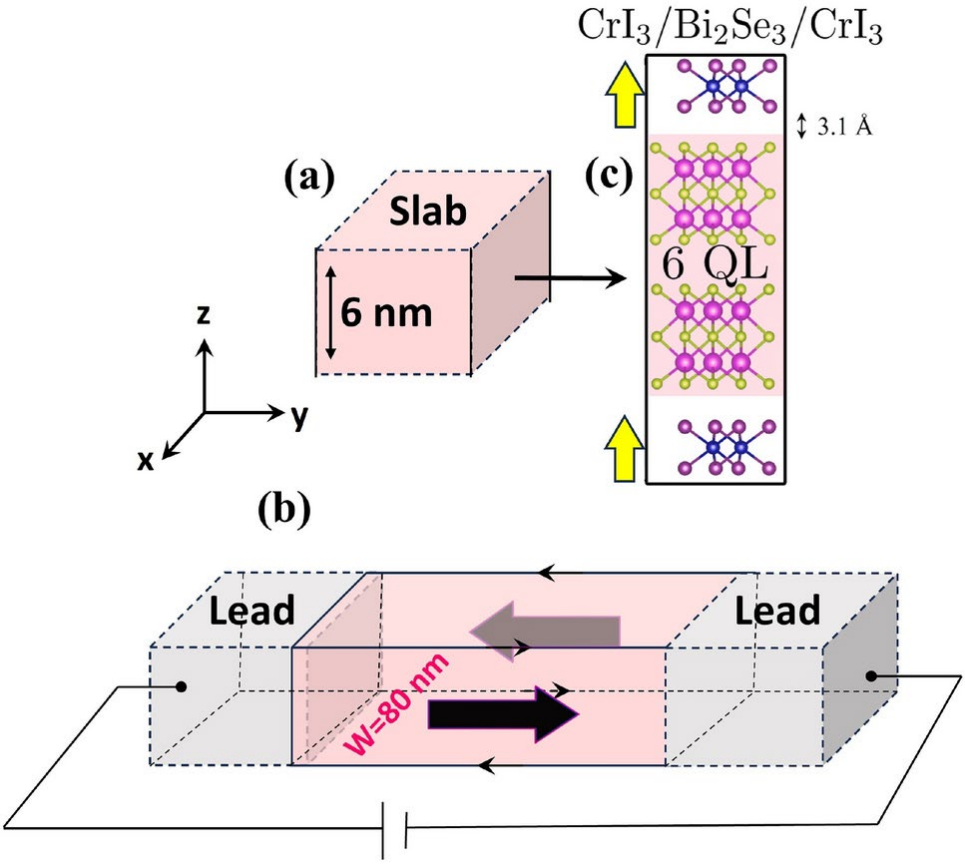
Schematic view of the CrI$_3$/Bi$_2$Se$_3$/CrI$_3$ heterostructure in (**a**) the slab geometry with 6 nm thickness and (**b**) the nanoribbon geometry where *w* is the width. The big black arrows represent the non-topological sidewall surface states due to the bulk states at higher Fermi energies and the small arrows show the chiral edge states that originate from the states inside the exchange gap. (**c**) The unit cell that we considered for the calculations. The yellow arrows show the direction of the magnetization at the interface.

Figure 1 provides a summarizing view of the system we address in the present work. Panel (a) illustrates the slab geometry. The slab we consider consists of 6 QL Bi$_2$Se$_3$, sandwiched between a top and bottom monolayer of CrI$_3$. The system is periodic in the *x*- and *y*-directions. In the *z*-direction it has thickness *d*. Surface states appear at the top and bottom surfaces of the slab, along the *xy*-plane. In panel (b), the nanoribbon geometry is outlined. This geometry is similar to the slab case, with the difference that the width *w* in the *x*-direction is now finite and the top and bottom surfaces are gapped due to the induced magnetization. The system is still periodic in the *y*-direction. The surfaces along the *yz*-plane of the nanoribbon are called sidewalls which host the surface states in the ribbon geometry, and the surfaces along the *xy*-plane are called top and bottom surfaces, in both the slab and nanoribbon geometry.

### Density functional calculations

To compute the electronic structure of the CrI$_3$/Bi$_2$Se$_3$/CrI$_3$ heterostructure, we have constructed a supercell in a slab geometry consisting of six quintuple layers (QLs) of Bi$_2$Se$_3$ sandwiched between structurally inversion-symmetric CrI$_3$ monolayers. To match the lattice constants one needs to consider a $\sqrt{3}\times \sqrt{3}$ supercell of Bi$_2$Se$_3$ in the lateral plane to match the CrI$_3$ monolayer^31^. We included a 15Å vacuum between adjacent slabs along the *z*-direction to avoid any couplings between them. Placing CrI$_3$ on top of the TI can be done in several ways. The configuration in which the Cr ions are placed on top of the Se atoms has earlier been found to energetically favorable^31^ and therefore we have selected this geometry in the present work. Thus, all self-consistent calculations in this work are performed using this setup. All DFT calculations are performed by employing the Vienna ab initio Simulation Package (VASP)^32,33^, with the Perdew-Burke-Ernzerhof generalized gradient approximation (PBE-GGA) for the exchange-correlation part of the energy functional^34^. We first optimized the crystal structure as regards the lattice parameters as well as the atomic positions using a *k*-mesh of size 6 $\times$ 6 $\times$ 1, until the stress on the cell and the average forces on the atoms became less than 0.02 eV/Å. The final relaxed structure was then used to study the electronic properties with the inclusion of the spin-orbit coupling (SOC); for this part we used a larger *k*-mesh of 12 $\times$ 12 $\times$ 1 to improve the accuracy of the calculations. To incorporate the effect of correlations at the transition metal Cr atoms, all self-consistent calculations were performed using GGA+U for the Cr atoms with effective on-site exchange interactions of 0.9 eV and effective on-site Coulomb interactions of 3 eV.

To analyze the topological properties of this system, we constructed a real-space Hamiltonian in the basis of the Wannier states for the low-energy bands. Constructing Wannier states of this complex system from DFT calculations is computationally very challenging, particularly with SOC. It takes both lots of time and memory to get the calculations done. We present these results in the result section. It is interesting to compare those results with alternative methods that require less time and memory allocation. For this reason, we also describe and analyze the topological and transport properties of the CrI$_3$/Bi$_2$Se$_3$/CrI$_3$ heterostructure using two model Hamiltonians, as explained below.

### Model hamiltonians

To analyze the electronic structure and transport properties of the CrI$_3$/Bi$_2$Se$_3$/CrI$_3$ heterostructure in the nanoribbon geometry, we employed a tight-binding model since DFT computations in the nanoribbon geometry quickly become prohibitely expensive.

In addition, we also used a four-band low-energy effective ${\varvec{k}}\cdot {\varvec{p}}$ Hamiltonian in momentum space to investigate the effect on the topological properties of the CrI$_3$/Bi$_2$Se$_3$/CrI$_3$ heterostructure in the slab geometry when the structural inversion symmetry between the top and bottom CrI$_3$ layers is broken.

Both models are described in further detail below.

#### Tight-binding model

To model the nanoribbon geometry, we employ a real-space tight-binding (TB) Hamiltonian, which enables simultaneous study of both the topological and transport properties in a nanoribbon geometry, see Fig. 1b.

In particular, we wish to study the effects of changing the width of the nanoribbon. The goal is to see the emergence of chiral edge states on the sidewalls of the nanoribbon as the system enters the CI phase.

We determine the parameters by fitting the parameters of the model to our DFT results, in this case specifically using the size of the gap. An attractive feature of this procedure is that it is computationally much more efficient than performing several first-principles calculations for different nanoribbons, which would in the end give qualitatively the same results.

The real-space TB Hamiltonian $H_C$ for the central part, i.e., the Bi$_2$Se$_3$ nanoribbon, is constructed as follows. We have first constructed a pristine Bi$_2$Se$_3$ slab of 6 QLs, then we have added an exchange field at the top and the bottom surface layers to mimic the effect of the CrI$_3$ magnetic monolayers on the Bi$_2$Se$_3$ surface states. The effect of this exchange field is to break TRS.

Since the strength of the exchange field determines the size of the gap at the Dirac point, we have chosen an exchange field that opens up a gap of 15 meV, i.e., the same gap size that we obtained from the DFT calculations.

We assume the following $sp^3$ TB model for Bi$_2$Se$_3$^35–37^:

1 $$\begin{aligned} \begin{aligned} H_C&= \sum _{ii',\sigma \alpha \alpha '}t_{ii'}^{\alpha \alpha '}e^{i{\varvec{k}}\cdot {\varvec{r}}_{ii'}}c_{i\alpha }^{\sigma \dag }c_{i'\alpha '}^{\sigma }\\ &\quad + \sum _{i,\sigma \sigma ',\alpha \alpha '} \lambda _i \left< i,\alpha ,\sigma |{\varvec{L}} \cdot {\varvec{S}}| i,\alpha ',\sigma ' \right> c_{i\alpha }^{\sigma \dag }c_{i\alpha '}^{\sigma '}\\ &\quad + \sum _{i,\sigma ,\alpha } M_i c_{i\alpha }^{\sigma \dag } {\sigma }_{z}c_{i\alpha }^{\sigma } . \end{aligned} \end{aligned}$$

In the first term, $t_{ii'}^{\alpha \alpha'}$ are the Slater−Koster parameters for the hopping energies. $c_{i\alpha }^{\sigma \dag }(c_{i\alpha }^{\sigma })$ is the creation (annihilation) operator for an electron with spin $\sigma$ in the atomic orbital $\alpha \in (s, p_x, p_y, p_z$) at atomic site *i*, ${\varvec{k}}$ is crystal momentum, and ${\varvec{r}}_{ii'}$ is the vector connecting site *i* and site $i'$.

$i^\prime \ne i$ runs over all neighbors of atom *i* in the same atomic layer as well as in the first and second nearest-neighbor layers in the adjacent cells In the second term, the on-site SOC is implemented in the intra-atomic matrix elements^38^, in which $\left| i,\alpha ,\sigma \right\rangle$ are spin- and orbital-resolved atomic orbitals. ${\varvec{L}}$ and ${\varvec{S}}$ are the orbital angular momentum and spin operators, respectively, and $\lambda _i$ is the SOC strength^35^. The last term is the exchange field with which we break TRS. It is applied only on the surface atoms. We assume $M=50$ meV, which yields a 15 meV surface gap.

#### Low-energy effective model

Since the topological nature of the CrI$_3$/Bi$_2$Se$_3$/CrI$_3$ slab is determined by the physics near the $\Gamma$ point, it is possible to obtain a meaningful characterization using a low-energy long-wavelength simple effective Hamiltonian. Therefore, we employ a low-energy effective so-called ${\varvec{k}}\cdot {\varvec{p}}$ model to describe and analyze the topological properties of the CrI$_3$/Bi$_2$Se$_3$/CrI$_3$ slab heterostructure, allowing us also to generalize to the case when structural inversion symmetry between the top and bottom CrI$_3$ layers is broken. The low-energy bands of the thin film of this system consist of only the Dirac-type surface states and the bulk states are always gapped. Therefore, the general effective ${\varvec{k}}\cdot {\varvec{p}}$ Hamiltonian can be written as^11^

2 $$\begin{aligned} \begin{aligned} \mathscr {H}_{{\varvec{k}}\cdot {\varvec{p}}}&= {\mathscr {H}}_{\textrm{surface}}+{\mathscr {H}}_{\textrm{Zeeman}}+{\mathscr {H}}_{\textrm{interface}} \\ \\&= \epsilon ({\varvec{k}})I_{4\times 4}+ \left[ \begin{array}{cccc} 0 & iv_F k_- & m({\varvec{k}}) & 0\\ -iv_Fk_+ & 0 & 0 & m({\varvec{k}})\\ m({\varvec{k}}) & 0 & 0 & -iv_Fk_-\\ 0 & m({\varvec{k}}) & iv_Fk_+ & 0 \end{array}\right] +\left[ \begin{array}{cccc} \Delta & 0 & 0 & 0\\ 0 & -\Delta & 0 & 0\\ 0 & 0 & \Delta & 0\\ 0 & 0 & 0 & -\Delta \end{array}\right] +\left[ \begin{array}{cccc} V_p & 0 & 0 & 0\\ 0 & V_p & 0 & 0\\ 0 & 0 & -V_p & 0\\ 0 & 0 & 0 & -V_p \end{array}\right] . \\ \\&=\left[ \begin{array}{cc} H_+(k) & V_p \sigma ^1 \\ V_p\sigma ^1 & H_-(k)\\ \end{array}\right] \end{aligned} \end{aligned}$$

This Hamiltonian is based on a four-band model considering the surface state dispersion ($\mathscr {H}_\textrm{surface}$), and incorporating the effects of magnetization ($\mathscr {H}_\textrm{Zeeman}$) which opens a magnetic gap at the Dirac point, and interfacial asymmetry ($\mathscr {H}_\textrm{surface}$). Here, the basis kets are $|t\uparrow \rangle$, $|t\downarrow \rangle$, $|b\uparrow \rangle$ and $|b\downarrow \rangle$, where *t* and *b* denote the top and bottom surface states and $\uparrow$, $\downarrow$ represent spin up and down states, respectively. The dispersion is modeled as $\epsilon ({\varvec{k}}) = A(k_x^2+k_y^2)$ where $\epsilon$ is the energy, ${\varvec{k}} = \left( k_x, k_y. k_z \right)$ is the crystal momentum, and *A* is a model parameter controlling the dispersion of the Dirac fermions determined from the fitting. In the second term, $v_F$ is the Fermi velocity and $k_{\pm } = k_x {\pm } ik_y$ are crystal momenta in the *xy*-plane. Continuing with the same term, $m({\varvec{k}})$ describes the tunneling effect between the top and bottom surface states of the Bi$_2$Se$_3$ slab and is given by $m({\varvec{k}})=M-B(k_x^2+k_y^2)$, where *M* and *B* are fitting parameters that control the quadratic correction in the coupling between surface states. In the third term, $\Delta$ is the exchange field along the *z*-axis introduced by the FM ordering emanating from the CrI$_3$ monolayers at the top and bottom surfaces. Here, $\Delta \propto \langle S\rangle$ with $\langle S\rangle$ being the mean-field expectation value of the local spin. The parameters of the effective model, i.e., *A*,*B*,*M*, $v_F$ and $\Delta$, are material-specific constants that determine the behavior of the Hamiltonian. They are obtained by fitting the energy spectrum of the effective Hamiltonian to the DFT-calculated bands near the $\Gamma$-point. Finally, we can put all terms in a compact format as indicated in the third line of Eq. 2 where $H_\pm (\textbf{k}) = v_F k_y \tau ^1 \mp v_F k_x \tau ^2 + \left( m(\textbf{k}) \pm \Delta \right) \tau ^3$, and $\tau ^i$ and $\sigma ^1$ are Pauli matrices.

When the slab thickness is very large, the top and bottom surface states are well separated spatially i.e $m({\varvec{k}})\approx 0$. However, as the film thickness is reduced, $m({\varvec{k}})$ becomes finite. An attractive feature of this model Hamiltonian above is that one can derive a simple condition, namely $\left| M\right| <\left| \Delta \right|$, that guarantees that the system is in the topological state, see^31^ for details.

Finally, in the last and fourth term in Eq. (2), $V_p$ represents the magnitude of the asymmetric interfacial potential that can occur in experiments due to misalignment of the top and bottom CrI$_3$ layers, and we keep it here as a parameter that can affect topological properties.

## Results

### Electronic properties

**Fig. 2:**
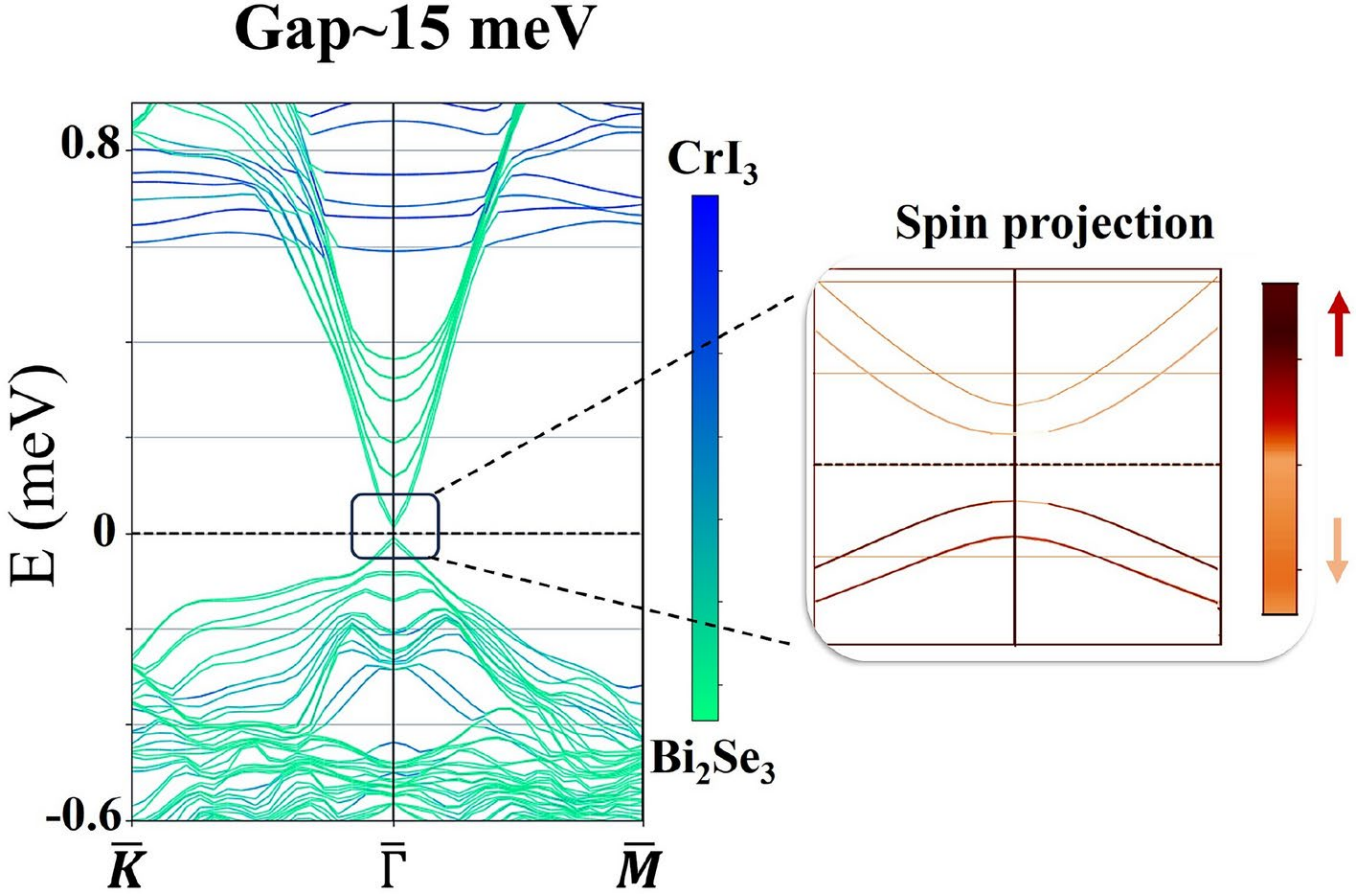
Projected band structure in the presence of spin-orbit coupling. The zoom-in figure shows the spin projection of the low energy bands.

Pristine Bi$_2$Se$_3$ is indeed gapless and exhibits a non-trivial band structure characterized by a Z$_2$ topological invariant, which features robust helical topological surface states protected by time-reversal symmetry. In the presence of an exchange field induced by CrI$_3$ in the vicinity of this topological insulator, an exchange gap opens up in the TI surface states, i.e. the Kramers degeneracy is lifted due to the breaking of time-reversal symmetry. The size of the gap depends on the magnitude of the magnetization induced as well as the coupling between adjacent layers in the heterostructure. Figure 1 shows the configuration we have considered. It consists of a 6QL Bi$_2$Se$_3$ sandwiched between two CrI3 monolayers. As is shown in Fig. 2, an induced exchange gap of 15 meV is derived from our DFT calculations.

**Fig. 3:**
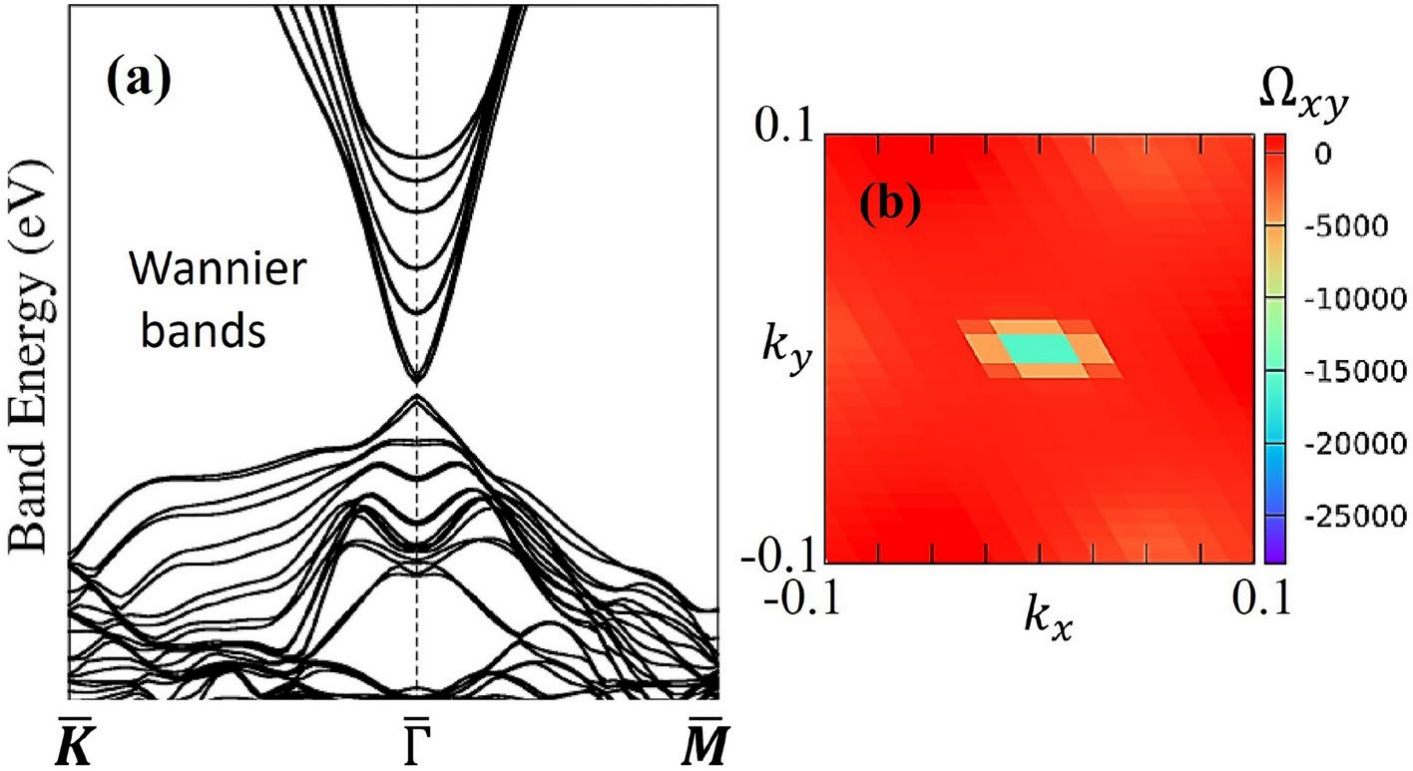
(**a**) Band structure derived from the Wannier function approach. (**b**) Berry curvature in the *xy*-plane.

To demonstrate that our system is indeed in the Chern insulator phase, we have constructed the Wannier functions with the basis set consisting of Bi and Se *p* orbitals and Cr *d* orbitals. As is shown in Fig. 3, the band structure based on this basis set closely resembles the one from the DFT calculations. Furthermore, post-processing analysis with WannierTools gives a non-zero Berry curvature in the *xy*-plane, which indicates that the system is in the topological phase.

### Phase diagram for a slab of magnetised TI

**Fig. 4:**
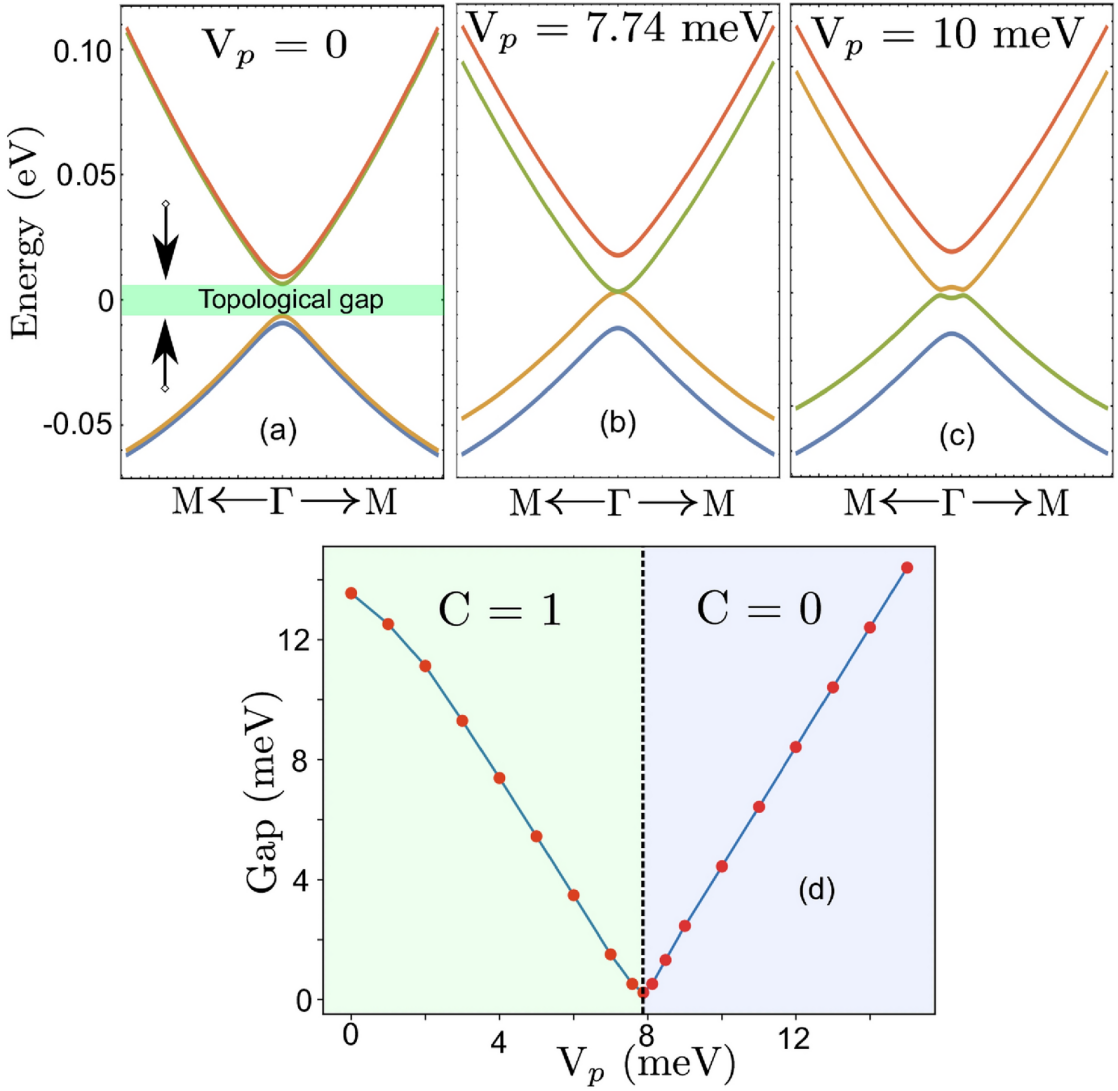
Band structure of CrI$_3$-Bi$_2$Se$_3$-CrI$_3$ heterostructure from low energy effective four band model increasing asymmetric interfacial potential $V_p$ with (**a**) $V_p$ = 0 meV, (**b**) $V_p$ = 7.74 meV and (**c**) $V_p$ = 10 meV respectively. (**d**) How the topological gap and Chern number varies as a function of $V_p$. A topological phase transition from a topological to a trivial insulator occurs at $V_p$ = 7.74 meV.

Following the ${\varvec{k}} \cdot {\varvec{p}}$ method described in Sect. 2.3, we fit the energy spectrum of the effective Hamiltonian using the DFT-calculated bands of the CrI$_3$/Bi$_2$Se$_3$/CrI$_3$ slab around the $\Gamma$ point. In this way, we obtain the fitting parameters $v_f = 2.395403$ eVÅ; $M = 0.001086$ eV; $B = 2.996947$ eVÅ$^2$; $\Delta = 0.007838$ eV; $A = 19.000841$ eVÅ$^2$. Using this result, we now further investigate the band topology of the system, following the rules that (i) it has Chern number $\mathcal C=1$ when $\Delta ^2 > M^2$ i.e in the topological phase and (ii) it has Chern number $\mathcal C = 0$ when $\Delta ^2 < M^2$ i.e., in the trivial phase^11^. These rules come about due to the fact that when $\Delta ^2 > M^2$, the exchange splitting $\Delta$ can overcome the coupling-induced gap *M*, resulting in a band inversion, which in turn gives rise to the non-trivial phase. In our setup, the CrI$_3$/Bi$_2$Se$_3$/CrI$_3$ slab has structural inversion symmetry, which gives the asymmetric interfacial potential $V_p = 0$. The parameter *M* depends on the thickness of the Bi$_2$Se$_3$ slab. Setting $M \sim 1$  meV, ww reproduce the slab band gap of $\sim 15$ meV computed from DFT. The corresponding energy spectrum for the CrI$_3$/Bi$_2$Se$_3$/CrI$_3$ slab is presented in Fig. 4a. In this phase, the CrI$_3$/Bi$_2$Se$_3$/CrI$_3$ slab is in the non-trival topological regime ($C = 1$), satisfying the condition $\Delta ^2 > M^2$.

Now, we slowly increase the asymmetric interfacial potential $V_p$ to explore the effect of breaking inversion symmetry, and study the evolution of the band structure of the CrI$_3$/Bi$_2$Se$_3$/CrI$_3$ slab, see panels (b) and (c) in Fig. 4. For $V_p < 7.74$ meV, the system must be in the topological phase since $\Delta ^2 > M^2 + V_p^2$. Thus, we have $C = 1$ in this regime. The topological gap at the $\overline{\Gamma }$ point closes as $V_p$ increases past 7.74 meV. This results first in a Dirac semi-metallic phase at the gap closing, i.e., when $\Delta ^2 = M^2 + V_p^2$. For $V_p > 7.74$ meV (i.e., $\Delta ^2 < M^2 + V_p^2$), the system undergoes a phase transition from from topological phase to a trivial phase ($C = 0$), illustrated in Fig. 4c. The corresponding phase diagram with respect to the asymmetric interfacial potential $V_p$ is shown in Fig. 4d. In summary, our analysis using this model indicates that the CrI$_3$/Bi$_2$Se$_3$/CrI$_3$ slab is in a topological insulating phase ($C=1$) with a topological gap at the $\overline{\Gamma }$ point for $V_p < 7.74$ meV, and transitions to a trivial insulator phase ($C=0$) for $V_p > 7.74$ meV.

### Topology in the ribbon geometry

**Fig. 5:**
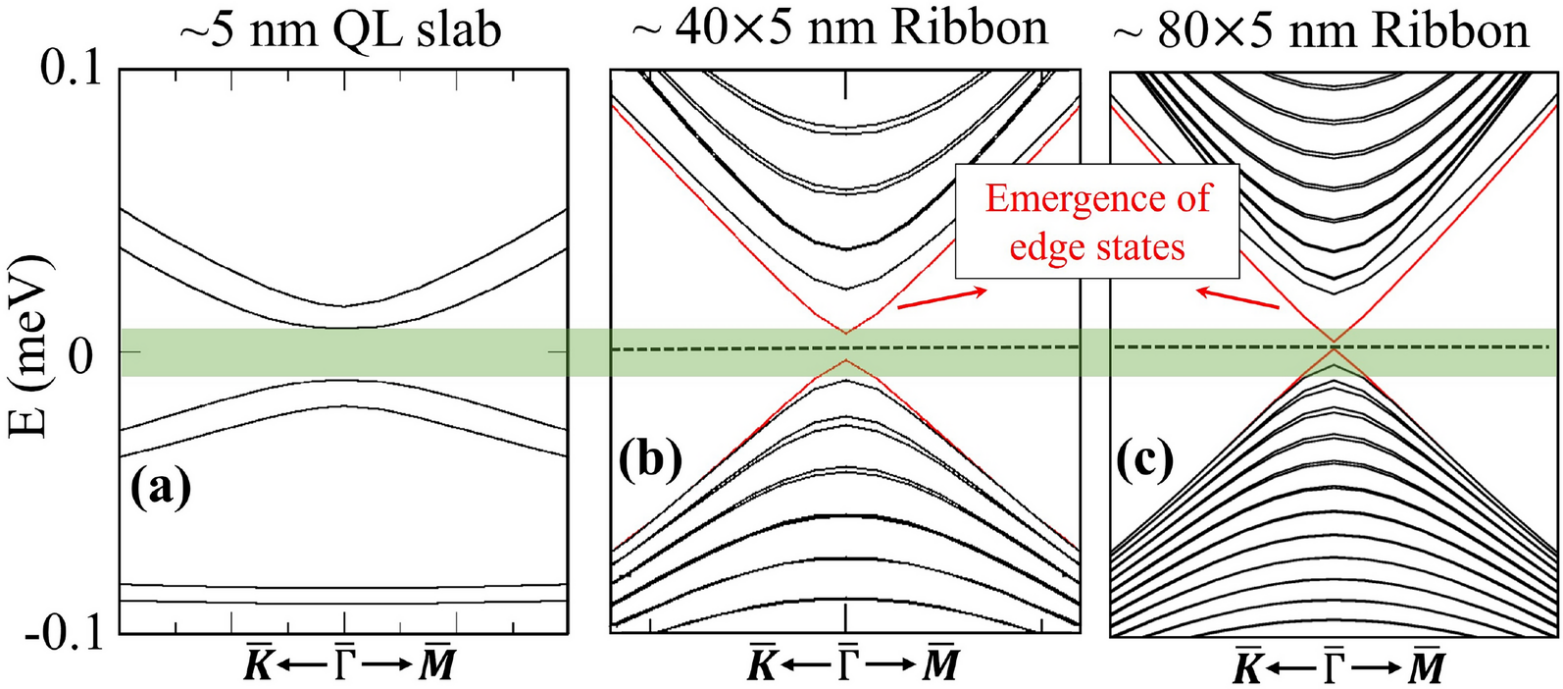
(**a**) Induced exchange gap at the Dirac point of the 6 QL heterostructure slab. (**b**) Emerging edge states in the 40 nm wide nanoribbon of a 6 QL slab. (**c**) Same as in panel (b), but for a 80 nm ribbon.

**Fig. 6:**
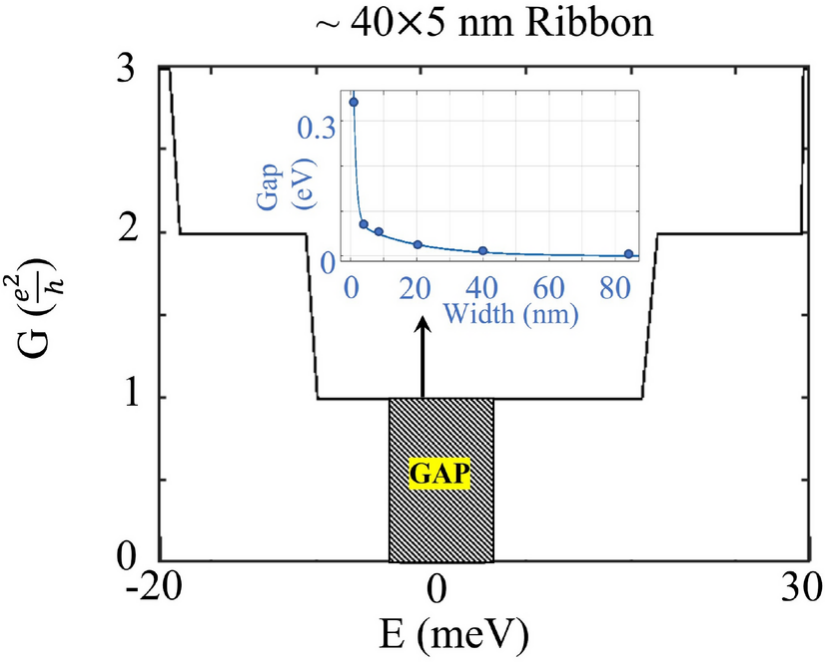
The spin conductance for a nanoribbon with 40 nm width and 6 QL. The inset shows that the size of the gap at the edge states exponentially reduces as the width of the ribbon is increased.

If we consider a nanoribbon by limiting the structure also in the *x* direction, as it is in the experimental setup, the edge states should appear inside the exchange gap. These edge states are chiral, meaning that they propagate in only one direction along the edge without backscattering. This behavior is a consequence of the non-trivial topology of the bulk material. In this configuration, $k_y$ is still a good quantum number and we can plot the band structure along this direction and study the emergence of the edge states. If we consider first the bulk geometry (infinite in all directions), there will be a bulk gap in the band structure as in all insulators. Now, if we restrict one dimension (along *z*) we obtain the slab configuration with surface states. In addition, there is an induced exchange gap due to the magnetization, as shown in Fig. 5a for the 6QL slab. In the next step, we make a nanoribbon by restricting the second dimension (along *x*) which causes the edge states to emerge inside the gap, see Fig. 5b. These edge states are spin polarized and chiral, which characterizes the Chern phase of the system. In this finite ribbon, having a small cross-section and a thin thickness leads to direct coupling of the surfaces, on the other hand, a thicker slab results in a higher contribution of the non-topological sidewall states which connect top and bottom surfaces. Therefore, even in the case of 6QL which does not have coupling between top and bottom surfaces, there is still a small gap in the band structure that prevents the formation of perfect edge states due to the coupling between the lateral side walls. Thus, the width should be larger than a critical value in order to see perfect quantization of the conductance, otherwise the edge states will still be gapped. By increasing the width of the system we can see from Fig. 5c that the gap shrinks and eventually vanishes. As is shown in the inset of Fig. 6, the size of the gap at the edge states decreases exponentially as the width of the nanoribbon is increased, and for the current setup minimum width of 80 nm gives a well-defined gapless edge states. If we compare this value with the minimum thickness along the *z* direction (which is about 25 nm) to have decoupled states and avoid opening the gap at the surface states, the width critical value is considerably larger and the reason is that the real-space distribution of the wave functions are broader for the side walls compared to the top and bottom surfaces.

In the next step, we look at the conductance and validate its quantized value. For this purpose we use the Green ’s functions to calculate conductance in the nanoribbon. Given the Hamiltonian of the central channel, the spin-dependent retarded (*r*) and advanced (*a*) Green’s functions are given by

3 $$\begin{aligned} \varvec{\mathcal{G}}^{r} (E)=[E^{+}\varvec{I}-\varvec{H}-\varvec{\Sigma }_{L}^{r}(E)- \varvec{\Sigma }_{R}^{r}(E)]^{-1}=[\varvec{\mathcal{G}}^{a}(E)]^{\dag }, \end{aligned}$$

where $\varvec{\Sigma } _{L(R)}(E)= \varvec{H}_{L(R),C}^{\dag } \varvec{g}_{L(R)}(E)\varvec{H}_{L(R),C}$ is the self-energy due to the connection of the left (right) semi-infinite electrode to the central channel. $\varvec{g}_{L(R)}$ is the surface Green’s function of the left (right) lead; $\varvec{g}_{R}$ is calculated iteratively by adding one unit cell from the central region at a time using the method of Ref.^39^. Therefore, the Green’s functions of a central region consisting of *M* unit cells each containing *N* atoms can be efficiently calculated using $2 N \times 2N$ matrices, where the factors of two come from the spin degree of freedom. $\varvec{H}_{L(R),C}$ is the tunneling Hamiltonian between the central region and the left (right) lead. The Hamiltonian of the leads, as well as the tunneling Hamiltonian, is identical to that of the central region, i.e., Eq. (1), except that it does not include the exchange field term. Now that we have the Green’s functions, the conductance can be calculated as

4 $$\begin{aligned} G(E) = \frac{e^{2}}{h}\textrm{Tr} [\varvec{\Gamma }^{L}(E) \varvec{\mathcal{G}}^{r} (E) \varvec{\Gamma }^{R} (E) \varvec{\mathcal{G}}^{a} (E)] \end{aligned}$$

where $\varvec{\Gamma }^{L,R} = i[ \varvec{\Sigma }^r_{L/R}- ( \varvec{\Sigma }^r_{L/R} )^\dag ]$ is the broadening due to the electrode contacts.

In Fig. 6, we show the calculated longitudinal conductance in the central channel. Due to the large matrices involved in this calculation, we have selected to perform the computation for just a few energy values, to elucidate the general behavior of the conductance. As is shown in this figure, the conductance has a stepwise shape with the first step at $G=\frac{e^2}{h}= G_0$, which confirms its quantized format. We interpret this value as having two $\frac{e^2}{2h}$, corresponding to each edge, i.e., considering both the top and bottom surfaces. The conductance remains constant at this quantized value when the Fermi energy is varied within the energy interval $[-8,19]$ meV. However, due to the coupling of the side-walls for this width, there will be a gap of 8 meV with no edge states. The energy interval corresponding to the first step is where only two chiral edge states are present. Outside this interval, other states intervene and the conductance increases.

## Conclusions

In summary, by breaking time-reversal symmetry through the proximity effect, we have analyzed the quantum anomalous Hall phase, which displays topological transport properties attributed to sidewall chiral edge states. Our study of nanoribbons within this context has revealed that the contribution of edge states depends critically on the system’s cross-section and thickness. The choice of these parameters governs the extent to which the non-trivial sidewall states, connecting the top and bottom surfaces, influence the material’s behavior. Specifically, we have examined a heterostructure comprising the topological insulator Bi$_2$Se$_3$, sandwiched between two-dimensional magnetic monolayers of CrI$_3$. Employing DFT we calculated the electronic band structure which then has been used as our principle to calculate topological and transport properties. Then we calculated the Wannier bands and showed that the system has a non-zero Berry curvature which confirms the topological phase. Since the size of the system is very large and the Wannier function calculations therefore is demanding in terms of computing time and memory allocation, we proposed an alternative way of topological analysis that can be done for the large systems in general. We used an effective ${\varvec{k}}\cdot {\varvec{p}}$ model that can be fitted to the low-energy states from the DFT results and gives a non-trivial Chern number for slabs thicker than 6QLs (30 nm). To verify the quantization of the conductance in a nanoribbon configuration which practically is related to a topological invariant (here the Chern number), we employed the non-equilibrium Green’s function method along with a tight-binding Hamiltonian which describes the system. Our investigations show that the width of the nanoribbon should be at least 80 nm (with thickness 30 nm) to see the perfect gapless edge states and quantized conductance. These findings contribute valuable insights into the design and understanding of materials with tailored topological and transport properties, and we hope that they will serve as a useful guide for further experimental studies in this field. In future research, it would be valuable to investigate heterostructures with varying thicknesses of CrI$_3$ to better understand how bulk-like properties influence the topological surface states of Bi$_2$Se$_3$. Such studies could reveal important insights into the magnetic interactions and interlayer coupling effects that arise in thicker CrI$_3$ layers.

## Data Availability

The data supporting the findings of this study are available in the paper and upon reasonable request from the corresponding author (N.P.).
